# Greater Short-Time Recovery of Peripheral Fatigue After Short- Compared With Long-Duration Time Trial

**DOI:** 10.3389/fphys.2020.00399

**Published:** 2020-05-14

**Authors:** Christian Froyd, Fernando G. Beltrami, Guillaume Y. Millet, Brian R. MacIntosh, Timothy D. Noakes

**Affiliations:** ^1^Faculty of Education, Arts and Sport, Western Norway University of Applied Sciences, Bergen, Norway; ^2^Division of Exercise Science and Sports Medicine, Department of Human Biology, University of Cape Town, Cape Town, South Africa; ^3^Exercise Physiology Lab, Institute of Human Movement Sciences and Sport, ETH Zurich, Zurich, Switzerland; ^4^Laboratoire Interuniversitaire de Biologie de la Motricité, University of Lyon, UJM Saint-Etienne, Saint Etienne, France; ^5^Human Performance Laboratory, Faculty of Kinesiology, University of Calgary, Calgary, AB, Canada

**Keywords:** peripheral fatigue, recovery, maximal voluntary contraction, femoral nerve electrical stimulation, motor unit recruitment, electromyography, self-paced exercise

## Abstract

The kinetics of recovery from neuromuscular fatigue resulting from exercise time trials (TTs) of different durations are not well-known. The aim of this study was to determine if TTs of three different durations would result in different short-term recovery in maximal voluntary contraction (MVC) and evoked peak forces. Twelve trained subjects performed repetitive concentric right knee extensions on an isokinetic dynamometer self-paced to last 3, 10, and 40 min (TTs). Neuromuscular function was assessed immediately (<2 s) and 1, 2, 4, and 8 min after completion of each TT using MVCs and electrical stimulation. Electrical stimulations consisted of single stimulus (SS), paired stimuli at 10 Hz (PS10), and paired stimuli at 100 Hz (PS100). Electrically evoked forces including the ratio of low- to high-frequency doublets were similar between trials at exercise cessation but subsequently increased more (*P* < 0.05) after the 3 min TT compared with either the 10 or 40 min TT when measured at 1 or 2 min of recovery. MVC force was not different between trials. The results demonstrate that recovery of peripheral fatigue including low-frequency fatigue depends on the duration and intensity of the preceding self-paced exercise. These differences in recovery probably indicate differences in the mechanisms of fatigue for these different TTs. Because recovery is faster after a 3 min TT than a 40 min TT, delayed assessment of fatigue will detect a difference in peripheral fatigue between trials that was not present at exercise cessation.

## Introduction

Self-paced endurance exercises of various intensities and durations lead to fatigue (Thomas et al., 2015). Already in 1891, it was suggested that fatigue has a central (the will) and a peripheral (muscle) origin and that sensation of fatigue is an important regulator of exercise performance (Mosso, 1915). Since then, several models have been developed to explain fatigue during exercise (Abbiss and Laursen, 2005). Recently, Noakes (2012) suggested that fatigue is a brain-derived emotion that regulates exercise performance. With some similarities to Noakes (2012) and Enoka and Duchateau (2016) suggest that fatigue has two attributes, performance and perceived fatigability. Perceived fatigability is defined as changes in the sensations that regulate the integrity of the performer. During exercise, perceived fatigability is derived from rates of change in the modulating factors like core temperature, motivation, and pain to regulate the pace and control the development of fatigue. Performance fatigability depends on the contractile capacities and activation of the working skeletal muscles (Kluger et al., 2013; Enoka and Duchateau, 2016).

The interaction between performance and perceived fatigability was evident in our pacing study, in which self-paced force, neuromuscular activation, and perceived exertion increased in the end-spurt despite decreasing levels of evoked forces (increasing peripheral fatigue) and possibly increasing levels of metabolites (Froyd et al., 2016a). Hence, a person may perform successfully despite a reduction in the ability to exert a maximal force (Enoka and Stuart, 1992), and afferent feedback from working muscles may not reduce neuromuscular activation (Froyd et al., 2016a).

A reduction in maximal voluntary contraction (MVC) force is often termed neuromuscular fatigue (Bigland-Ritchie and Woods, 1984). Neuromuscular fatigue can be further divided into peripheral and central fatigue, in which central fatigue is defined as a reduction in the maximal capacity of the central nervous system to recruit motor units to produce force or to discharge at a sufficient frequency (Gandevia, 2001). Peripheral fatigue is defined as a reduction in force originating from sites at or distal to the neuromuscular junction (Gandevia, 2001). To determine the types of fatigue, measurements of MVC force and surface electromyography (EMG) have been used, coupled with evoked muscle contractions (Millet et al., 2011). A reduction in evoked peak force responses to electrical stimulation (single or double pulses) are reported as evoked peak force and is a measure of peripheral fatigue (Place et al., 2010). Low-frequency fatigue can be measured by dividing response to low-frequency by that to higher-frequency electrical stimulation (Verges et al., 2009).

The current knowledge on the origins of peripheral fatigue suggests that exercise could impair three main components: action potential transmission along the sarcolemma, excitation–contraction coupling and actin–myosin interaction (Millet et al., 2011). Low-frequency fatigue is usually associated with a failure in the excitation–contraction coupling, due to a reduction in Ca^2+^ release from the sarcoplasmic reticulum (Hill et al., 2001) and possibly due to decreased myofibrillar Ca^2+^ sensitivity (Bruton et al., 2008).

Neuromuscular fatigue, measured as a reduction in MVC and evoked peak forces, increased with increasing exercise time during a self-paced time trial (TT) (Froyd et al., 2016a) and during exercise at a fixed intensity (Froyd et al., 2018). However, it is uncertain whether MVC and evoked peak forces are reduced more after short compared with longer duration self-paced TTs of various locomotor exercises (Carroll et al., 2017). The reason is that only two studies have directly compared MVC and evoked peak forces that developed during self-paced dynamic endurance exercises of different durations (Thomas et al., 2015; Froyd et al., 2016a). In those studies, MVC force was reduced to similar levels at exercise cessation in all trials, and central fatigue was greater after longer compared with shorter TTs involving either single-leg dynamic knee extensions (Froyd et al., 2016a) or cycling (Thomas et al., 2015), However, peripheral fatigue was greater after the shorter (4 vs. 40 km) cycling trials (Thomas et al., 2015), whereas after single-leg dynamic knee-extension TTs, peripheral fatigue was greater after the longest (40-min) compared with shortest (3-min) trial (Froyd et al., 2016a).

After cessation of self-paced exercise, MVC, and evoked peak forces, including the ratio of force with low-frequency stimulation to that of high-frequency stimulation increases substantially within a minute (Froyd et al., 2013). However, there is absence of knowledge about the recovery of fatigue following trials of different durations (Carroll et al., 2017). This is an important issue, as different rates of recovery of MVC and evoked peak forces might point toward different mechanisms underlying these different measures of fatigue. Peripheral fatigue recovery is associated with Ca^2+^-related mechanisms. [Ca^2+^] in myoplasm is decreased, probably because of impairment of Ca^2+^ release from the sarcoplasmic reticulum. In addition, submaximal force production is influenced by phosphorylation of the regulatory light chains of myosin (RLC). The increase in RLC phosphorylation during exercise, causing increased sensitivity to Ca^2+^, enhances submaximal contractile response (Rassier and Macintosh, 2000). This potentiation, which has the opposite effect of peripheral fatigue, coexists with peripheral fatigue both during exercise and in the early phase of the recovery processes after exercise (Rassier and Macintosh, 2000), although dephosphorylation of RLC occurs within the first few minutes after exercise cessation (Macintosh et al., 2012).

Comparison of peripheral fatigue in studies of different exercise durations, especially immediately after locomotor exercises like running, cycling, and skiing, present methodological problems because assessments are usually performed only 1–2 min after exercise cessation (Carroll et al., 2017). This delay is probably sufficiently long for peripheral fatigue to recover substantially and hence to affect the measured fatigue at the end of exercise and during recovery (Froyd et al., 2013, 2014). In addition, it is not known how exercise duration influences the interaction between fatigue and potentiation during recovery and how delayed assessments differ between trials of different durations and intensities.

Accordingly, the aim of this study was to measure and compare the time courses of short-term recovery in MVC and evoked peak forces after self-paced exercise of three different durations. To address this aim, subjects performed concentric self-paced knee extension exercise on an isokinetic dynamometer, allowing assessment of MVC and evoked peak forces, using voluntary and electrically evoked contractions immediately (<2 s) and 1, 2, 4, and 8 min after completion of the TTs. We hypothesized that evoked peak force would increase more, relative to pre-exercise, for short compared with long TT and that this difference would be apparent within the first 1–2 min following exercise cessation.

## Materials and Methods

The present study is the second part of an experiment investigating neuromuscular fatigue and self-paced TTs of different durations. Detailed description of the procedures for warm-up and the experimental TTs are described in full in Froyd et al. (2016a).

### Subjects

Twelve subjects (11 males, mean ± SD age 24.0 ± 6.3 years, body mass 75.0 ± 11.4 kg, and height 179.0 ± 9.7 cm) trained in both endurance and strength (training > 7 times a week) participated in the study. Subjects with previous or current injuries of the right leg were excluded. The study was approved by the Regional Ethics Committee Vest in Norway (2012/1486) and the Research and Ethics Committee of the Faculty of Health Science of the University of Cape Town, and the experiments were performed according to the latest (2013) revision of the Declaration of Helsinki. Subjects were instructed to refrain from high-intensity exercise on the day prior to testing and to refrain from alcohol during the last 24 h before testing. Subjects were also instructed to eat a light meal 2–4 h before arrival at the laboratory. Written informed consent was obtained from all subjects who participated in the study. Subjects were given a full explanation of the details and rationale of the study and were informed that they were free to withdraw at any time. The possibility that electrical stimulation might cause discomfort was fully explained, as was the nature of the risks involved.

### Experimental Design

**FIGURE 1:**
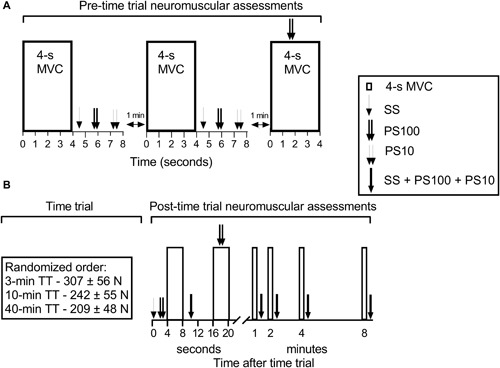
Schematic view of the protocol. **(A)** The neuromuscular function assessed prior to the TTs. MVCs were performed three times with a 1-min break between MVCs. SS, PS100, and PS10 were assessed after the first two MVCs, and PS100 (superimposed doublet) was applied during the third MVC. **(B)** The three time trials in randomized order, followed by assessment of neuromuscular function (MVC + electrical stimulation after 4–8 s and 1, 2, 4, and 8 min, MVC with superimposed doublet after 16–20 s). MVC, maximal voluntary contraction; SS, single stimulus; PS100, paired stimuli at 100 Hz; PS10, paired stimuli at 10 Hz; TT, time trial.

Subjects made nine visits to the laboratory, including six familiarization and three experimental trials separated by 2–3 days. During all familiarization trials, the participants were subjected to neuromuscular function (NMF) assessment and performed a self-paced TT with the right leg, which was always the dominant leg on the dynamometer (Kinematic Communicator, Chattecx Corp., Chattanooga, TE) (Figure 1). During visits 1–6, subjects performed two familiarization trials similar to each of the three experimental trials. The experimental TTs of short, middle, and long durations (visits 7–9) were performed in randomized order. NMF was assessed prior to, during, and after the TTs. Subjects received constant visual feedback of their force production from a computer screen during TTs and NMF assessments.

### Settings

**FIGURE 2:**
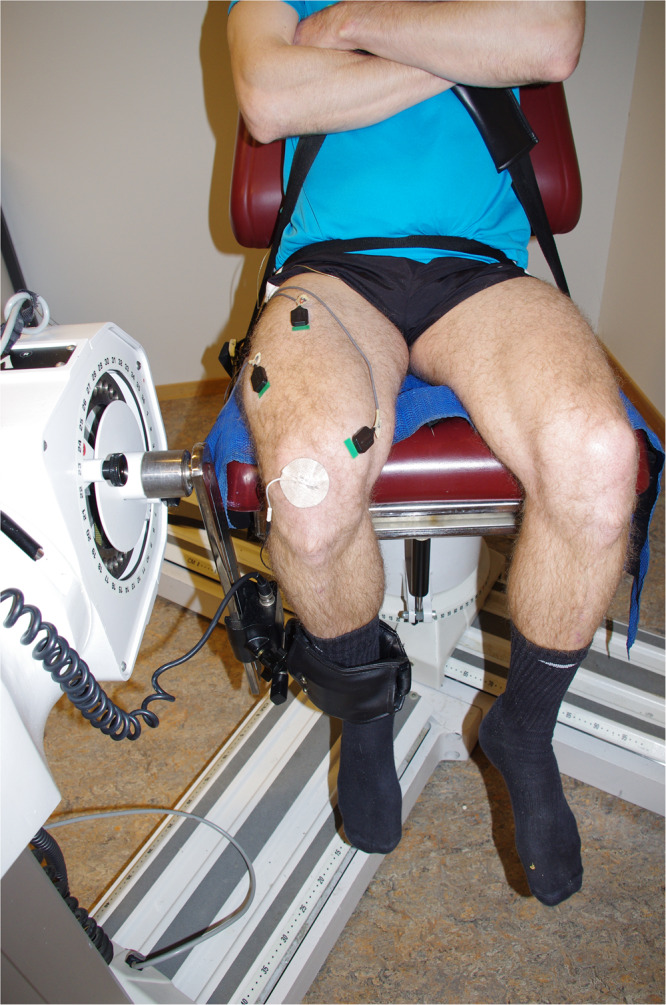
Photo of a subject secured to the dynamometer. Electromyography (EMG) of surface sensors and the reference sensor applied to the patella is visible.

Subjects were secured to the dynamometer by chest and hip straps to avoid excessive movements (Figure 2). The seating was adjusted for each subject, with the right femoral epicondyle aligned with the axis of rotation of the dynamometer. The right lower leg was attached to the lever arm just above the lateral malleolus. The seat’s backrest was reclined 10 degrees; the dynamometer’s rotation arm was kept at 90 degrees, and hip angle and right leg knee angles were ~110 and 80 degrees, respectively, during NMF assessments. The left leg was not active at any time and was secured to the dynamometer by a strap around the upper leg. Subjects kept their hands crossed in front of their upper body and maintained the same position during all experiments.

### Pre-Time Trial and Post-Time Trial Neuromuscular Function Assessments

Two minutes following concentric and isometric warm-up, and five maximal concentric contractions, NMF assessment (Figure 1A) started. NMF assessment consisted of a 4-s isometric MVC, followed immediately (<2 s) by a sequence of electrical stimulation on the relaxed muscles. During the MVCs, subjects were instructed to reach maximum force within 1 s and then to maintain that level of contraction effort for 3 s while they received strong verbal encouragement. Femoral nerve electrical stimulation consisted of a single stimulus (SS), paired stimuli at 10 Hz (PS10), and paired stimuli at 100 Hz (PS100). The interval between sequential stimulations was 1.5 s.

Pre-TT NMF consisted of three MVCs with a 1-min break between MVCs. SS, PS100, and PS10 were performed after the first two MVCs. PS100 (superimposed doublet) was also applied when the subject produced maximal force during the third MVC. Owing to this sequence of activations, it is assumed that SS, PS100, and PS10 were similarly potentiated for all measures (pre-TT and post-TT).

Post-TT NMF (Figure 1B) (4-s MVC + SS, PS100, and PS10) was assessed immediately (<2 s) and 1, 2, 4, and 8 min after completion of the TTs. In addition, an MVC with superimposed doublet was assessed immediately (<5 s) after the first NMF assessment after TT cessation.

### Experimental Time Trial

The TTs started 5 min after the end of the pre-TT NMF assessment. During each self-paced TT, the participant was encouraged to complete the TTs with the highest possible average force. The subjects received constant visual feedback of their force production and how much of the TT they had completed. Each TT consisted of sets of 15 concentric contractions, and sets were separated by breaks of 5 or 10 s during which subjects either rested or underwent NMF assessments. The numbers of sets were 4 for short, 16 for middle, and 64 for long TT, resulting in TT duration of 3, 10, and 40 min, respectively. Range of motion was from 90 to 15 degrees of knee flexion (0 degree is full extension), and each contraction consisted of a knee extension phase at 60 deg⋅s^–1^ and a passive knee flexion phase at 120 deg⋅s^–1^. The 15th concentric contraction was maximal for the following sets; 3-min TT, 1–4; 10-min TT, 1–4, 6, 8, 10, 12, and 14–16; and 40-min TT, 1–4, 6, 8, 12, 16, 24, 32, 40, 48, 56, 60, and 62–64. A 10-s NMF assessment period including isometric MVC and sequence of electrical stimulation (Figure 1A) followed after the following sets; 3-min TT, 1–4; 10-min TT, 1–4 and 14–16; and 40-min TT, 1–4 and 62–64.

### Data Collection

A high-voltage (maximal voltage 400 V) constant current stimulator (DS7AH, Digitimer, Hertfordshire, United Kingdom) triggered by a Power Lab, and Lab Chart software (ADInstruments Pty Ltd., Bella Vista, Australia) was used to deliver square-wave stimuli of 1-ms duration. The femoral nerve was stimulated percutaneously via a 10-mm-diameter self-adhesive cathode electrode (Skintact, Austria) pressed manually by the investigator onto the skin at the femoral triangle. The triangle is a subfacial space, which appears as a depression when the thigh is flexed. The anode, a 130 × 80-mm self-adhesive electrode (Cefar-Compex Scandinavia AB, Sweden) was applied to the gluteal fold. The stimulation intensity for one SS was determined by increasing the current gradually from 5 mA until a plateau in force (15–35 mA) and maximal M-wave amplitudes were reached. The current was then increased by 30% to ensure supramaximal stimulation. The stimulation current was kept constant for the same subject for all types of electrical stimulation.

Force in response to electrical stimulation, as well as voluntary force (isometric and concentric), was detected by a load cell mounted on the lever arm of the dynamometer. The reliability of the dynamometer has been described elsewhere (Mayhew et al., 1994). Force from the dynamometer and simultaneous EMG signals were recorded with Power Lab (ADInstruments). Force was digitally filtered using a low-pass filter at 50 Hz in Lab Chart Pro software, version 7.3.8 (ADInstruments).

EMG signals from the vastus lateralis (VL) and vastus medialis (VM) of the right leg were recorded using DE-2.1 single differential surface sensors with 10-mm inter-electrode distance (Delsys Inc., Boston, MA, United States). Delsys recommendations were used for the placement of the sensors on the skin. Sensors were placed in a direction parallel to the general direction of the skeletal muscle fibers. The reference electrode was applied to the patella. EMG signals were sampled at 2,000 Hz, amplified (gain = 1,000) using Bagnoli-8 (Delsys Inc.), and filtered using a band-pass filter with a bandwidth of 15–500 Hz in Lab Chart Pro software, version 7.3.8 (ADInstruments).

### Data Analysis

MVC force was measured for 1 s during the period of peak force development, that is, 500 ms before and after peak force. The largest MVC force measured before the trials was taken as the pre-TT MVC. The peak force responses to electrical stimulation are reported as evoked peak force, and a reduction in evoked peak force is a measure of peripheral fatigue. Evoked peak force for PS10/PS100 was calculated as an index of low-frequency fatigue (Verges et al., 2009).

The root mean square (RMS) of the EMG data of VL and VM was calculated for 1 s during the period of peak force of the MVC, that is, 500 ms before and after peak force. M-wave peak-to-peak amplitude (PPA) in response to SS was assessed. RMS during the MVCs was normalized by dividing it with the M-wave PPA of the following SS to estimate neuromuscular activation (RMS⋅M^–1^) (Millet et al., 2011). Because of the similarities, results for M-wave and RMS⋅M^–1^ are presented as an average of VL and VM data (Froyd et al., 2016a). In several studies, RMS⋅M^–1^ has been used to calculate neuromuscular activation (Froyd et al., 2016a, b; Torres-Peralta et al., 2016), whereas RMS has been used to calculate the central motor drive (Amann and Dempsey, 2008).

### Statistical Analyses

All analyses were performed using GraphPad Prism, v8.02 (La Jolla, CA, United States). The results are presented as mean ± SD unless otherwise noted. After the normality of data distribution was checked using the Shapiro–Wilk test, two-way repeated-measures ANOVAs with Tukey *post-hoc* assessments were used to detect differences over time (pre vs. post 0, 1, 2, 4, and 8 min) and between distances (3, 10, and 40 min TT), [time × condition]. Where the assumption of sphericity (Mauchly’s test) was violated, the Greenhouse–Geisser epsilon correction was applied to the degrees of freedom. Statistical tests for the different parameters over the recovery period were performed with values expressed as a function of baseline or end-exercise values. Comparisons of absolute values were performed only between baseline and end-exercise data (Sidak’s *post-hoc*, where applicable). Effect sizes for the different ANOVA effects were calculated as eta squared, representing the proportion of total variance explained by the factor (range 0.0–1.0). The statistical significance was defined at *P* < 0.05.

## Results

Absolute values pre-exercise and at exercise cessation, and after 1–8 min of rest are presented in Table 1. Briefly, MVC and evoked force responses that have already been presented elsewhere (Froyd et al., 2016a) were reduced after all TTs (main effect of time, all *P* < 0.0001) without time × trial interactions (Table 1). In contrast, MVC RMS⋅M^–1^, which was decreased after the 40-min TT only [*F*_interaction_ (1.345, 13.45) = 6.147, *P* = 0.02, η^2^ = 0.064], when it was lower than the values following the 3-min TT (*P* = 0.02). M-wave PPA decreased following the 40-min TT (*P* = 0.0003), whereas it increased after the 3-min TT (*P* = 0.02), so that values at cessation of exercise were different between all trials [*F*_interaction_ (1.780, 17.80) = 28.49, *P* < 0.0001, η^2^ = 0.156].

**TABLE 1:** Neuromuscular function for each distance at pre-exercise; at the end of TT; and after 1, 2, 4, and 8 min of recovery.

**Parameter**	**TT (min)**	**Pre-exercise**	**End of TT**	**Recovery**			
				**1 min**	**2 min**	**4 min**	**8 min**
MVC (N)	3	825 ± 147	555 ± 113****	659 ± 155	686 ± 165	721 ± 169	749 ± 165
	10	794 ± 153^##^	504 ± 91****^##^	627 ± 135	660 ± 128	696 ± 138	721 ± 146
	40	801 ± 153^#^	522 ± 116****^#^	669 ± 134	681 ± 135	702 ± 118	738 ± 130
MVC RMS⋅M^–1^ VL + VM	3	0.074 ± 0.012	0.086 ± 0.027	0.073 ± 0.013	0.068 ± 0.012	0.068 ± 0.01	0.079 ± 0.017
	10	0.075 ± 0.012	0.082 ± 0.022	0.075 ± 0.021	0.072 ± 0.011	0.069 ± 0.012	0.079 ± 0.012
	40	0.082 ± 0.019	0.072 ± 0.016*^#^	0.08 ± 0.022	0.073 ± 0.012	0.068 ± 0.009	0.073 ± 0.015
SS (N)	3	195 ± 49	85 ± 2****	147 ± 30	167 ± 36	164 ± 37	147 ± 35
	10	189 ± 46	80 ± 24****	131 ± 22	138 ± 25	136 ± 24	126 ± 25
	40	191 ± 42	78 ± 30****	129 ± 27	130 ± 27	124 ± 27	116 ± 25
PS10 (N)	3	310 ± 67	135 ± 44****	222 ± 49	256 ± 56	253 ± 60	229 ± 58
	10	299 ± 64	125 ± 37****	194 ± 33	205 ± 40	197 ± 37	181 ± 39
	40	303 ± 62	120 ± 51****	179 ± 47	184 ± 50	173 ± 49	163 ± 43
PS100 (N)	3	298 ± 67	185 ± 40****	247 ± 49	263 ± 54	262 ± 57	248 ± 57
	10	290 ± 60	176 ± 27***	233 ± 35	237 ± 38	232 ± 38	224 ± 42
	40	296 ± 59	167 ± 37****	226 ± 37	225 ± 37	219 ± 39	217 ± 40
FPS10/FPS100	3	1.05 ± 0.10	0.72 ± 0.14****	0.90 ± 0.13	0.98 ± 0.12	0.97 ± 0.12	0.93 ± 0.14
	10	1.03 ± 0.08	0.71 ± 0.16****	0.84 ± 0.12	0.97 ± 0.14	0.86 ± 0.14	0.81 ± 0.14
	40	1.03 ± 0.09	0.70 ± 0.18****	0.79 ± 0.15	0.81 ± 0.15	0.78 ± 0.15	0.75 ± 0.13
PPA VL + VM (mV)	3	3.95 ± 0.32	4.13 ± 0.24*	4.16 ± 0.28	4.17 ± 0.27	4.00 ± 0.26	3.78 ± 0.23
	10	4.01 ± 0.35	3.71 ± 0.34^#^	3.86 ± 0.31	3.84 ± 0.29	3.69 ± 0.31	3.56 ± 0.35
	40	3.94 ± 0.24	3.35 ± 0.42***^#$^	3.46 ± 0.47	3.41 ± 0.49	3.33 ± 0.48	3.31 ± 0.49

Values are expressed in absolute units ± SD (n = 12, except n = 11 for MVC RMS⋅M^–1^ and PPA). MVC, maximal voluntary contraction; RMS, root mean square; M, M-wave; VL, vastus lateralis; VM, vastus medialis; SS, single stimulus; PS10, paired stimuli at 10 Hz; PS100, paired stimuli at 100 Hz; FPS10/FPS100, ratio of force for PS10 vs. PS100; PPA, peak-to-peak amplitude of the M-wave; TT, time trial. *Different from respective pre-exercise. ^#^Different from 3-min TT. ^$^Different from 10-min TT. The number of symbols indicate significance at *P < 0.05; ***P < 0.001; ****P < 0.0001. Comparisons are presented for pre-exercise and end of TT only.

**FIGURE 3:**
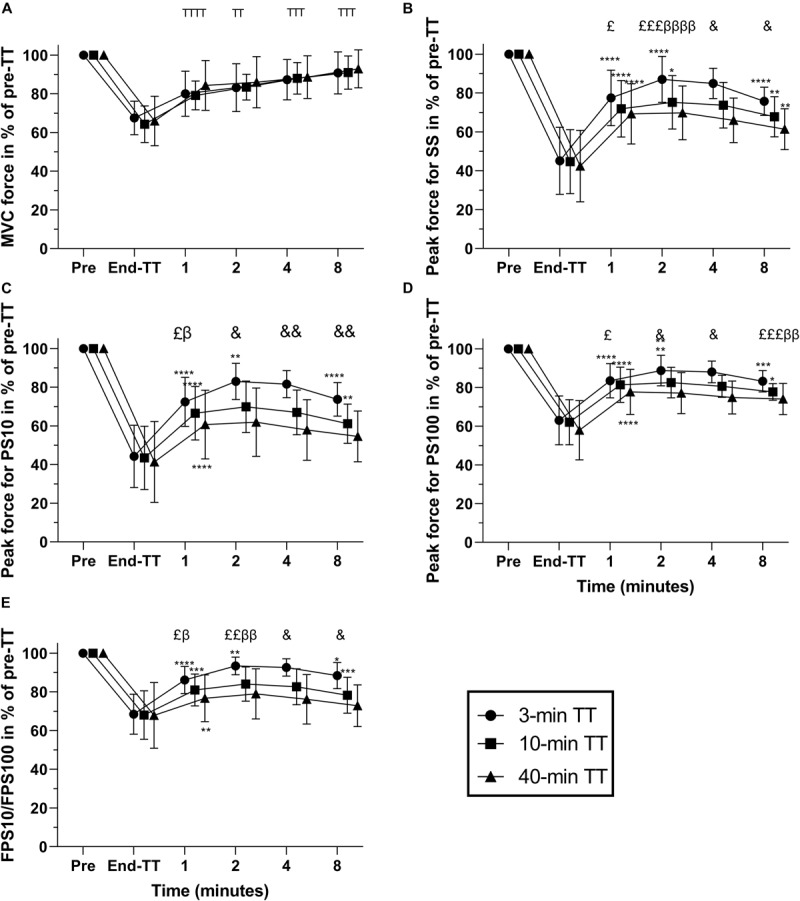
Responses in% of pre-TT for 3, 10, and 40 min duration TTs pre-TT; at the end of TT; and during the first 1, 2, 4, and 8 min following exercise cessation. MVC (maximal voluntary contraction) force **(A)**, peak force for SS (single stimulus) **(B)**, peak force for PS10 (paired stimuli at 10 Hz) **(C)**, peak force for PS100 (paired stimuli at 10 Hz) **(D)**, and FPS10/FPS100 (force for paired stimuli at 10 Hz divided by that for 100 Hz) **(E)**. Data are shown as means ± SD, *n* = 12. ^*T*^Significant difference from previous, main effect of time. *Significant difference from previous. ^β^ Significant difference between 3 min TT and 10-min TT. ^£^Significant difference between 3 min TT and 40 min TT. ^&^Significant difference between all TTs’ β, *P* < 0.05. The number of symbols indicate significance at *P < 0.05; **P < 0.01; ***P < 0.001; and ****P < 0.0001. Numerical data for these parameters is provided in Supplementary Table S1. TT, time trial.

The different fatigue-related parameters expressed as% change from the end-TT values are presented in Table 2. There was a main effect of time for MVC force, with MVC already 24.1 ± 5.5% higher than at end-TT in the first minute of recovery (*P* < 0.001 vs. end-TT). MVC force recovery was not different between the TTs during the 8-min rest period and increased at each subsequent interval (Figure 3). Evoked peak force responses to SS, PS10, and PS100 increased significantly (all *P* < 0.001) during the first minute after exercise cessation for all TTs relative to values measured at end of TT (Table 2). Evoked peak force responses to all types of electrical stimulation (SS, PS10, and PS100), as well as the ratio of force for PS10/PS100, increased more (*P* < 0.05) for the 3 min TT compared with either the 10-min or 40-min TT after 2 and up to 8 min of exercise cessation.

**TABLE 2:** Neuromuscular function during recovery as percentage change from end of TT.

Parameter	TT duration (min)	1 min	2 min	4 min	8 min	*F*_*time*_ (DFn, DFd), *P*, η^2^ *F*_*trial*_ (DFn, DFd), *P*, η^2^ *F*_interaction_ (DFn, DFd), *P*, η^2^
MVC force Δ%	3	18 ± 6	23 ± 9**	30 ± 9****	35 ± 9***	*F* (1.249, 13.74) = 35.50, *P* < 0.0001, η^2^ = 0.160
	10	25 ± 15	32 ± 16**	39 ± 16****	44 ± 2***	*F* (1.668, 18.35) = 3.064, *P* = 0.0786, η^2^ = 0.066
	40	29 ± 14	32 ± 13**	37 ± 16****	44 ± 20***	*F* (4.202, 46.22) = 0.9511, *P* = 0.4464, η^2^ = 0.004
MVC RMS⋅M^–1^ (VL + VM) Δ%	3	−11 ± 16	−18 ± 15	−17 ± 15	−5 ± 17***	*F* (1.344, 13.44) = 6.306, *P* = 0.0189, η^2^ = 0.048
	10	−8 ± 14	−10 ± 12	−13 ± 16	−1 ± 18***	*F* (1.673, 16.73) = 7.637, *P* = 0.0061, η^2^ = 0.146
	40	12 ± 20^###$$$^	3 ± 18^####$$$^	−2 ± 19^####$$$^	4 ± 19***^####$$$^	*F* (3.735, 37.35) = 1.700, *P* = 0.1739, η^2^ = -0.020
Force for SS Δ%	3	82 ± 32	108 ± 46**	106 ± 54	84 ± 46****	*F* (1.047, 11.51) = 10.28, *P* = 0.0074, η^2^ = 0.030
	10	70 ± 29^#^	79 ± 35*^##^	77 ± 38^##^	64 ± 40**^##^	*F* (1.449, 15.94) = 4.887, *P* = 0.0309, η^2^ = 0.056
	40	78 ± 43	81 ± 48	73 ± 50^#^	62 ± 51*	*F* (2.324, 25.57) = 9.647, *P* = 0.0005, η^2^ = 0.017
Force for PS10 Δ%	3	72 ± 28	101 ± 43**	100 ± 49	79 ± 41***	*F* (1.066, 11.72) = 11.50, *P* = 0.0050, η^2^ = 0.039
	10	61 ± 25	70 ± 28*^##^	64 ± 32^##^	50 ± 31**^##^	*F* (1.573, 17.30) = 13.64, *P* = 0.0006, η^2^ = 0.125
	40	61 ± 32	65 ± 38^##^	56 ± 41^##^	49 ± 40^##^	*F* (2.546, 28.01) = 10.01, *P* = 0.0002, η^2^ = 0.018
Force for PS100 Δ%	3	35 ± 14	44 ± 19**	43 ± 22	35 ± 20***	*F* (1.071, 11.78) = 4.152, *P* = 0.0627, η^2^ = 0.015
	10	33 ± 12	35 ± 13^#^	33 ± 16^#^	28 ± 18*	*F* (1.581, 17.40) = 2.097, *P* = 0.1592, η^2^ = 0.023
	40	39 ± 23	38 ± 22	35 ± 25	33 ± 26	*F* (2.956, 32.51) = 8.615, *P* = 0.0003, η^2^ = 0.011
FPS10/FPS100 Δ%	3	27 ± 10	39 ± 17**	38 ± 18	31 ± 14*	*F* (1.116, 12.27) = 15.55, *P* = 0.0015, η^2^ = 0.047
	10	21 ± 11	25 ± 12^##^	23 ± 11^##^	16 ± 9***^##^	*F* (1.900, 20.90) = 20.33, *P* < 0.0001, η^2^ = 0.257
	40	16 ± 13^##^	19 ± 13^###^	15 ± 13^##^	10 ± 14^###^	*F* (3.432, 37.75) = 3.676, *P* = 0.0165, η^2^ = 0.015
PPA (VL + VM) Δ%	3	1 ± 3	1 ± 6	−3 ± 5**	−8 ± 4**	*F* (1.648, 16.48) = 20.90, *P* < 0.0001, η^2^ = 0.279
	10	4 ± 3^##^	4 ± 4	0 ± 4**	−4 ± 3*^#^	*F* (1.751, 17.51) = 3.195, *P* = 0.0711, η^2^ = 0.062
	40	3 ± 2^#^	2 ± 5	−1 ± 5	−2 ± 8^#^	*F* (3.335, 33.35) = 4.584, *P* = 0.0069, η^2^ = 0.031

Values are expressed as percentage change from end of TT (n = 12, except n = 11 for MVC RMS⋅M^–1^ VL and PPA VL). MVC, maximal voluntary contraction; RMS, root mean square; M, M-wave; VL, vastus lateralis; VM, vastus medialis; SS, single stimulus; PS10, paired stimuli at 10 Hz; PS100, paired stimuli at 100 Hz; FPS10/FPS100, ratio of force for PS10 vs. PS100; PPA, peak-to-peak amplitude of the M-wave. *Different from previous. ^#^Different from 3-min TT. ^$^Different from 10-min TT. The number of symbols indicate significance at *P < 0.05; **P < 0.01; ***P < 0.001; ****P < 0.0001; ^##^P < 0.01; ^###^P < 0.001; ^####^P < 0.0001; ^$$$^P < 0.001.

Evoked peak force responses to all types of electrical stimulation (SS, PS10, and PS100) in percentage of pre-exercise were not different between the trials at exercise cessation, but 1 min after exercise cessation, evoked peak forces had already increased more after the 3 min TT compared with after the 40-min TT (mean difference + SE for SS 8.2 ± 2.5 percent points, *P* = 0.018; PS10 11.8 ± 2.7 percent points, *P* = 0.0026; PS100 5.8 ± 1.9 percent points, *P* = 0.0247; Figure 3). After 2 min of recovery, evoked responses were also higher after the 3-min TT compared with the 10 min TT (SS 11.8 ± 2.2 percent points, *P* = 0.0007; PS10 13.2 ± 2.1 percent points, *P* = 0.0001; PS100 6.3 ± 1.5 percent points, *P* = 0.0247). These differences persisted until 8 min after the TT. The force ratio for PS10/PS100, a measure of low-frequency fatigue, was also not different between the trials at exercise cessation, but already 1 min after exercise cessation, the ratio had increased more after the 3-min TT compared with both the 10-min (5.2 ± 1.5, *P* = 0.013) and 40-min TT (9.5 ± 2.0, *P* = 0.001), and the difference was also sustained throughout the evaluated recovery period.

**FIGURE 4:**
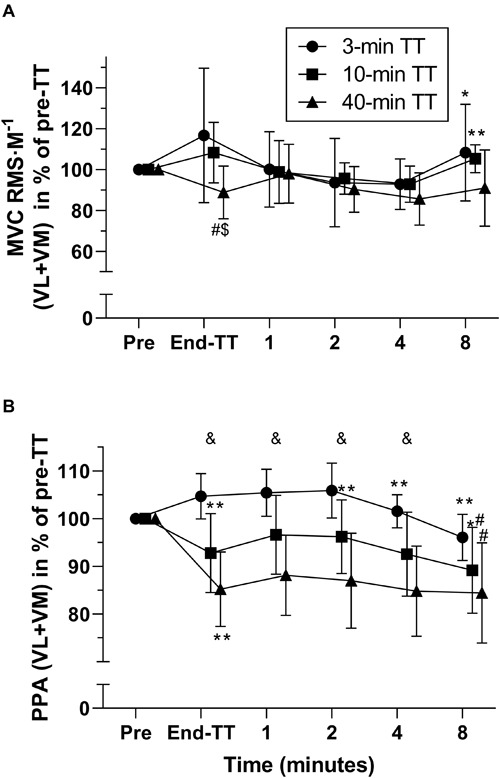
Electromyography (EMG) responses during recovery, expressed as % of pre-TT for 3, 10, and 40 min duration TTs. MVC RMS normalized to M-wave amplitude (average vastus lateralis and vastus medialis **(A)**. M-wave peak-to-peak amplitude (average vastus lateralis and vastus medialis) expressed as % of pre-TT **(B)**. Data are shown as means ± SD, *n* = 11. MVC, maximal voluntary contraction; RMS, root mean square; M, M wave; PPA, peak-to-peak amplitude; VL, vastus lateralis; VM, vastus medialis. *Different from previous. ^#^Different from 3-min TT. ^&^All three TTs are different from each other at this time point. The number of symbols indicate significance at *P < 0.05 and **P < 0.01.

MVC RMS⋅M^–1^ in percentage of pre-exercise was significantly lower [*F*_interaction_ (4.668, 46.68) = 3.637, *P* = 0.0085, η^2^ = 0.052] after the 40-min TT than after 10-min (-19.5 ± 5.4 percent points, *P* = 0.0128) and 3-min TT (-27.9 ± 9.5 percent points, *P* = 0.0365), but this difference was not significant after 1-min recovery (Figure 4). M-wave PPA at exercise cessation was reduced significantly more [*F*_interaction_ (3.365, 33.65) = 6.604, *P* = 0.0009, η^2^ = 0.013] for the 40-min compared with 10-min TT (-7.6 ± 2.5 percent points, *P* = 0.0296), which in turn was also lower than the 3-min TT (-11.9 ± 2.5 percent points, *P* = 0.002). These differences persisted until the 8th minute of recovery, when the 40-min TT was no longer different from the 10-min TT (-4.8 ± 2.1 percent points, *P* = 0.1089).

## Discussion

The main aim of the present study was to compare the short-term recovery in MVC and evoked peak forces between self-paced TTs of three different durations. The most important findings of this study were as follows:

i. Evoked peak forces (SS, PS10, and PS100) recovered more after the 3-min TT compared with the 40-min TT.
ii. The ratio for force for PS10/PS100, an index of low-frequency fatigue, recovered more after the 3-min TT compared with the 40 min TT.
iii. MVC force continued to improve throughout recovery whereas evoked responses improved only for the first 1–2 min after exercise cessation.

At the cessation of exercise, the similar evoked peak force between the TTs can be explained by a relatively similar end-spurt in force. Unpublished data collected during the TTs showed that self-paced force during the last four sets (3 min) of the TTs were 64, 61, and 59% of the pre-exercise values for 3, 10, and 40 min TT, respectively. Additionally, M-wave PPA, an index of neuromuscular propagation (Fuglevand et al., 1993), was more reduced at exercise cessation of the short compared with the longer TT, but these differences were constant throughout the recovery period. Therefore, neuromuscular transmission or excitation of the sarcolemma was probably not influencing recovery of NMF in any substantial way. Hence, recovery of neuromuscular fatigue is probably mostly due to alterations distal to the sarcolemma.

Despite similar values at exercise cessation, evoked peak forces recovered more, within the first minute after exercise cessation, following the short compared with the longer TT, providing strong evidence that the rate of the recovery processes is influenced by the duration–intensity characteristics of the preceding self-paced exercises. This is in accordance with what we hypothesized based on comparison of previous studies, specifically that peripheral fatigue persists for a long time after long distance endurance exercise (Carroll et al., 2017), whereas it recovers more quickly after shorter exercises, for example, an isometric sustained MVC (Vernillo et al., 2018) or a 6 min TT (Froyd et al., 2013). Given that force production was relatively similar at the end of all trials, these differences in rates of recovery are likely to result from the demands of the exercise preceding the final four sets of repetitions during the longer trials. Hence, similarity in the end-spurt between trials may have caused a similar level of peripheral fatigue at exercise cessation, whereas the differences in exercise duration may have contributed to differences in recovery of peripheral fatigue between trials.

The recovery of SS-evoked force (~80%) was greater than that of the recovery of PS100-evoked force (~35%) 1 min after exercise cessation (Table 2). Therefore, although PS100-evoked force was less reduced than the force for SS or PS10 after 1 or 2 min of recovery, SS- and PS10-evoked forces recovered more during this time. Indeed, unpublished data from our group show that potentiated evoked peak force to an SS increased more (46 ± 26%, *P* < 0.001) in 1 min than the response to high-frequency doublets (100 Hz) (21 ± 20%) (Froyd et al., 2013). This observation is in contrast with a report by Carroll et al. (2017); the time course of force recovery of forces evoked by high-frequency stimulation (e.g., PS100) is more rapid than is force recovery measured with low-frequency stimulation (SS of PS10).

At exercise cessation, reduction in force for SS or PS10 is expected to be greater than the reduction in force for PS100 (Allen et al., 2008b), and we have confirmed this observation both in the present experiment (Froyd et al., 2016a) and in our previous studies (Froyd et al., 2013, 2014, 2016b, 2018). A larger decrease in responses to low vs. high-frequency stimulation is present in all situations in which low-frequency stimulation occurs on the steeper part of the force–[Ca^2+^]_*i*_ relationship than is the case with high-frequency stimulation. Thus, all situations in which [Ca^2+^]_*i*_ and/or the myofibrillar Ca^2+^ sensitivity are reduced have the potential of showing low-frequency fatigue (Allen et al., 2008b). It is possible that force responses to low-frequency stimulation are reduced by both [Ca^2+^]_*i*_ and myofibrillar Ca^2+^ sensitivity, whereas force responses to high-frequency stimulation are reduced more by [Ca^2+^]_*i*_ (Allen et al., 2008a) or by a reduced force production per cross-bridge (Edman and Lou, 1990) (i.e., reduced contractility).

Greater recovery in evoked peak forces including FPS10/FPS100 after the 3-min compared with 40-min TT is a novel and interesting finding, as it indicates that different mechanisms may contribute to peripheral fatigue depending on the duration of self-paced exercise trials. Mechanisms might be related to metabolic response (Bogdanis et al., 1995; Macintosh and Shahi, 2011) and differences in muscle glycogen depletion (Allen et al., 2008b; Green et al., 2011). A combination of those factors could contribute to a similar level of peripheral fatigue between trials at exercise cessation, but a different level of peripheral fatigue during recovery. Early stages of fatigue involve impaired myofibrillar function that has two components: decreased ability of the actomyosin cross-bridge to generate force and reduced myofibrillar Ca^2+^ sensitivity. The third component that becomes more involved in later stages of exercise is reduced SR Ca^2+^ release. Impaired myofibrillar function is probably not related to lactate, creatine, and H^+^ (i.e., reduced pH or acidosis) (Cheng et al., 2018; Olsson et al., 2020). Cytoplasmic ATP concentration is relatively constant during most types of exercise (Cheng et al., 2018) and, therefore, is not supposed to induce fatigue in any of the trials. Instead, increased inorganic phosphate (Pi) concentration is proposed as the dominant cause of reduced muscle contractility (Cheng et al., 2018), which in turn will regulate activation to reduce ATP breakdown (Macintosh and Shahi, 2011). Increased Pi during exercise (typically 1–2 min of duration) will contribute to both impaired myofibrillar function and SR Ca^2+^ release (Macintosh and Shahi, 2011; Cheng et al., 2018). There may be greater changes in Pi after short and intense compared with longer and less intense exercise (Spriet et al., 2000), whereas muscle glycogen concentrations may be lower after longer and less intense compared with shorter and more intense exercise (Areta and Hopkins, 2018). SR Ca^2+^ release can become reduced with more prolonged and lower intensity exercise in relation to reduced glycogen stores in the muscle fibers (Cheng et al., 2018), and this could be the case for the 40-min vs. 3-min TT. Because muscle metabolites like Pi can recover significantly in 90 s without any recovery in glycogen concentrations (Bogdanis et al., 1995), recovery of Pi could explain the difference in rates of recovery of peripheral fatigue between trials. In addition, increased reactive oxygen/nitrogen species can also cause reduced SR Ca^2+^ release, resulting in prolonged reduction in contractility after exercise (Bruton et al., 2008; Cheng et al., 2016) similar to all three TTs in the present study.

It is also possible that recovery of evoked forces could be masked by different degrees of potentiation at different time points. At cessation of all TTs, it is probable that phosphorylation of the myosin RLC was maximal or close to maximal in the muscles that were important for this exercise. When stimulating before and after the MVC at exercise cessation (Figure 1B), evoked peak force for SS increased by 7% after the 3 min TT and by 2% after the 40 min TT. Hence, the MVC did not lead to much additional potentiation, as was expected (Froyd et al., 2014). Decay of evoked peak forces after 4 and 8 min, on the other hand, might be partly explained by a loss of potentiation – less phosphorylation at the time of measurement of the evoked responses. Although potentiation dissipates over the first few minutes of inactivity, recovery from fatigue proceeds. In our experiments, potentiation would have been maintained at the time the evoked responses were obtained (after the MVC), thus negating any impact of changes in potentiation on the measured evoked responses. Considering that low-frequency contractions are more affected by potentiation than high-frequency contractions, this fact would reduce the chances of observing low-frequency fatigue.

Interestingly, although peak evoked forces showed a different recovery pattern between trials of different durations, this was not the case for forces generated by the MVC. Because central fatigue was present at the cessation of the 40-min TT (voluntary activation reduced by 4%), but not after the 3-min TT (Froyd et al., 2016a), a potential recovery of central fatigue – as indicated by increased neuromuscular activation (RMS⋅M^–1^) during the MVC performed after the 40 min TT (Table 2) – could, to some extent, explain why MVC force was not different between trials, whereas evoked peak forces recovered more after the 3 min compared with 40 min TT. Nevertheless, voluntary activation seems not to recover during such a short period as 8 min (Kruger et al., 2019).

Although many studies have assessed peripheral fatigue two or more minutes after termination of a TT (Amann and Dempsey, 2008; Girard et al., 2012; Thomas et al., 2015), we have shown that peripheral fatigue begins to recover within 1–2 min after termination of exercise (Froyd et al., 2013, 2014). Delayed assessments of peripheral fatigue cause underestimation of peripheral fatigue and can explain the higher levels of peripheral fatigue reported in our TT studies (Froyd et al., 2013, 2016a) compared with other studies of similar duration (Amann and Dempsey, 2008; Girard et al., 2012; Thomas et al., 2015). If we had performed the first assessment of peripheral fatigue after 2 min, we would have reported no recovery of fatigue during the next 6 min (Figure 3), and our results would be relatively similar to those reported after cycling TTs (Amann and Dempsey, 2008; Thomas et al., 2015). Because a delay of 1–2 min is long enough to obscure the observation of fatigue at the end of exercise and the recovery kinetics, one solution for this can be to use a special type of cycling ergometer in which fatigue can be measured immediately after exercise cessation (Doyle-Baker et al., 2018). By using this ergometer, Kruger et al. (2019) found more peripheral fatigue after a 10-min compared with 90-min trial but without any recovery during the first 2 min after those trials. These results contrast with our TT results (Figure 3), possibly owing to methodological differences because subjects in those trials cycled at fixed intensities, without exercising to exhaustion (Kruger et al., 2019) or with an end-spurt. The end-spurt increases peripheral fatigue during TTs (Froyd et al., 2016a).

We have reported rapid recovery in peripheral fatigue after a 6-min TT (Froyd et al., 2013, 2014). However, now, we extend this finding to show that early recovery is rapid for TTs lasting from 3 to 40 min. Possible differences in low-frequency fatigue and potentiation in the recovery period between trials of different durations can lead to erroneous conclusions with respect to the magnitude of fatigue if there is a delay in the initial force measurements after the task causing the fatigue. For instance, assessing peripheral fatigue 4 min after exercise may result in differences between trials; however, those differences may be the result of different recovery processes and not due to the exercise itself. Hence, the underestimation of peripheral fatigue would be greater for a 3-min compared with 40-min TT.

### Methodological Considerations

This study was performed in a laboratory with single-joint exercise on a dynamometer, which allowed us to assess fatigue within 2 s after exercise cessation. Hence, we were able to identify recovery in NMF already 1 min after exercise cessation. However, the results cannot be generalized to activities, such as running or cycling. Voluntary activation was not assessed in the recovery period. This measurement could provide additional valuable information and could explain the similarities in MVC force recovery and differences in peripheral fatigue recovery between trials. Instead, we used RMS⋅M^–1^ to calculate neuromuscular activation. RMS⋅M^–1^ is not considered as valid as voluntary activation to describe changes in central drive (Place et al., 2007). However, amplitude cancelation that increases owing to fatigue limits the association between EMG amplitude and the intensity of the neural activation (Farina et al., 2014).

Central regulation (Noakes, 2012) and perceived fatigability (Kluger et al., 2013; Enoka and Duchateau, 2016) probably influenced performance during the trials and, therefore, could influence the recovery process after the trials. We did not control for factors like motivation, expectations, mental fatigue, and pain perception. However, the three trials were randomized, subjects performed two familiarization sessions for each trial to improve experience, and we recorded rating of perceived exertion, which was not different between trials. MVC and peak evoked forces during recovery are mainly related to contractile function (Neyroud et al., 2012).

## Conclusion

MVC and evoked peak forces recovered substantially within 1 min after completion of TTs lasting from 3 to 40 min. MVC continued to improve throughout recovery, whereas evoked responses improved only for the first 1–2 min. Peripheral fatigue, measured as both evoked peak forces and PS10/PS100, an index of low-frequency fatigue, recovered more after the 3-min TT compared with the 40-min TT. The results demonstrate that recovery of peripheral fatigue including low-frequency fatigue depends on the duration and intensity of the preceding self-paced exercise. These differences in recovery probably indicate differences in the mechanisms of fatigue for these different TTs. Because recovery of peripheral fatigue is greater after a 3-min TT than a 40-min TT, delay in assessment of fatigue will result in a difference in peripheral fatigue observed between trials that was not present at exercise cessation.

## Data Availability Statement

The datasets generated for this study are available on request to the corresponding author.

## Ethics Statement

The study was approved by the Regional Ethics Committee Vest in Norway (2012/1486) and the Research and Ethics Committee of the Faculty of Health Science of the University of Cape Town, and the experiments were performed according to the latest (2013) revision of the Declaration of Helsinki. The subjects were not allowed to consume alcohol or stimulants during the 24 h preceding the exercise tests. Written informed consent was obtained from all subjects who participated in the study.

## Author Contributions

CF and TN conceptualized and designed the study. CF collected the data and analyzed the data. CF, FB, GM, BM, and TN interpreted the data, revised the manuscript, approved the final version of the manuscript, and agreed to be accountable for all aspects of the work in ensuring that questions related to the accuracy or integrity of any part of the work are appropriately investigated and resolved. CF drafted the manuscript.

## Conflict of Interest

The authors declare that the research was conducted in the absence of any commercial or financial relationships that could be construed as a potential conflict of interest.

## Abbreviations

EMG: electromyography
MVC: maximal voluntary contraction
NMF: neuromuscular function
PPA: peak-to-peak amplitude of the M-wave
PS10: paired stimuli at 10 Hz
PS100: paired stimuli at 100 Hz
FPS10/FPS100: ratio of evoked peak force for PS10 vs. PS100
RLC: regulatory light chains of myosin
RMS: root mean square
RMS ⋅ M^–1^: root mean square/M-wave peak-to-peak amplitude
SS: single stimulus
TT: time trial
VL: vastus lateralis
VM: vastus medialis.
