# Supplementary material for: Greater Short-Time Recovery of Peripheral Fatigue After Short- Compared With Long-Duration Time Trial
Source: Front Physiol. 2020 May 14;11:399. doi: 10.3389/fphys.2020.00399 (PMC7240104; doi:10.3389/fphys.2020.00399)
Supplement: Supplementary file 1 [file Table_1.DOCX]

**Table S1.** Neuromuscular function for each TT-distance as percentage (100% = no change) of pre-TT, at the end of TTs and after 1, 2, 4 and 8 minutes of recovery.

| **Parameter** | **TT-distance (min)** | **End of TT** | **Rest** | | | |
| --- | --- | --- | --- | --- | --- | --- |
|  |  |  | **1 min** | **2 min** | **4 min** | **8 min** |
| MVC force  Δ% | 3 | 68 ± 9 | 80 ± 12 | 83 ± 12 | 87 ± 10 | 91 ± 11 |
|  | 10 | 64 ± 9 | 79 ± 7 | 83 ± 7 | 88 ± 8 | 91 ± 9 |
|  | 40 | 66 ± 13 | 84 ± 13 | 86 ± 13 | 89 ± 11 | 93 ± 10 |
| MVC RMS∙M^−1^  (VL+VM) Δ% | 3 | 117 ± 33 | 100 ± 18 | 94 ± 22 | 93 ± 12 | 108 ± 24 |
|  | 10 | 108 ± 15 | 99 ± 15 | 96 ± 8 | 93 ± 9 | 105 ± 7 |
|  | 40 | 89 ± 13 | 98 ± 14 | 90 ± 11 | 86 ± 13 | 91 ± 19 |
| Force for SS  Δ% | 3 | 45 ± 17 | 77 ± 14 | 87 ± 12 | 85 ± 8 | 76 ± 7 |
|  | 10 | 45 ± 16 | 72 ± 15 | 75 ± 14 | 74 ± 12 | 68 ± 10 |
|  | 40 | 42 ± 18 | 69 ± 16 | 70 ± 14 | 66 ± 11 | 61 ± 11 |
| Force for PS10  Δ% | 3 | 44 ± 16 | 72 ± 13 | 83 ± 9 | 82 ± 7 | 74 ± 9 |
|  | 10 | 43 ± 16 | 67 ± 14 | 70 ± 13 | 67 ± 12 | 61 ± 10 |
|  | 40 | 41 ± 21 | 61 ± 18 | 62 ± 18 | 58 ± 16 | 55 ± 13 |
| Force for PS100  Δ% | 3 | 63 ± 13 | 84 ± 9 | 89 ± 8 | 88 ± 6 | 83 ± 6 |
|  | 10 | 62 ± 12 | 81 ± 9 | 83 ± 8 | 81 ± 6 | 78 ± 4 |
|  | 40 | 58 ± 15 | 78 ± 12 | 77 ± 11 | 75 ± 9 | 74 ± 8 |
| FPS10/FPS100  Δ% | 3 | 68 ± 10 | 86 ± 7 | 93 ± 5 | 93 ± 4 | 88 ± 7 |
|  | 10 | 68 ± 12 | 81 ± 8 | 84 ± 9 | 83 ± 9 | 78 ± 9 |
|  | 40 | 68 ± 17 | 77 ± 12 | 79 ± 13 | 76 ± 13 | 73 ± 11 |
| PPA (VL+VM)  Δ% | 3 | 105 ± 5 | 105 ± 5 | 106 ± 6 | 102 ± 3 | 96 ± 5 |
|  | 10 | 93 ± 8 | 97 ± 8 | 96 ± 8 | 93 ± 9 | 89 ± 9 |
|  | 40 | 85 ± 8 | 88 ± 8 | 87 ± 10 | 85 ± 9 | 84 ± 10 |

Data are shown as means ± SD (*n* = 12, except *n*= 11 for MVC RMS∙M^−1^ and PPA). MVC, maximal voluntary contraction; RMS, root mean square; M, M-wave; VL, vastus lateralis; VM, vastus medialis; SS, single stimulus; PS10, paired stimuli at 10 Hz; PS100, paired stimuli at 100 Hz; FPS10/FPS100, ratio of force for PS10 vs PS100; PPA, peak to peak amplitude of the M-wave. Please refer to the main text and figure 2 of the manuscript for statistical differences.
